# Abdominal pain in hypertriglyceridemia

**DOI:** 10.1093/gastro/gou001

**Published:** 2014-01-30

**Authors:** Khalid Alkimawi

**Affiliations:** Tufts Medical Center, Department of Gastroenterology, Boston, MA

**Keywords:** acute pancreatitis, plasmapharesis, hypertriglyceridemia

## BACKGROUND

Hypertriglyceridemia increases the risk of acute pancreatitis, accounting for a minor but clinically relevant proportion of cases. We present a case of diabetic ketoacidosis, complicated by severe hypertryglyceridemia and pancreatitis. The pancreatitis was managed and controlled by managing the hypertryglyceridemia. We aim to review the specific management targeted for this subset of the population.

## CLINICAL CASE

We report a 41-year-old male patient known to have diabetes mellitus type two (DM II) and hypertension (HTN). He was being treated with metformin and atenolol for DM II and HTN, respectively. He presented to our emergency department with nausea, bilious vomiting and abdominal pain, which was mainly central and had back radiation. This had had a sudden onset and progression over the previous 24 hours. He did not have any previous surgeries to report and denied consuming alcohol or using recreational drugs. On his examination, he was only noted to have central abdominal tenderness. His lab. work showed white blood count (WBC) 14.1/mm^3^, Na (Sodium) 128 meq/L, K (Potasium) 5.0 meq/L, Cl (Chloride) 92 meq/L, bicarbonate 9 meq/L, anion gap 27, glucose 722 mg/dL, lipase 7687 U/L. His liver enzymes, bilirubin and albumin were all normal. A right upper quadrant ultrasound scan showed a normal gall bladder, no echogenic stones, normal caliber bile ducts and normal hepatopedal flow. The cardiac nature of this pain was excluded; his electrocardiogram (ECG) and cardiac enzymes were normal. The triglyceride was checked and was found to be 4851 U/L. At that point, the patient was admitted to our intensive care unit. Diabetic ketoacidosis was managed with intravenous fluids, insulin drips and electrolyte repletion. The next day, the patients anion gap closed but he continued to have abdominal pain and vomiting, the patient underwent plasmapherisis for four hours. The triglycerides went down to 532 U/L after one cycle and symptoms improved.

## DISCUSSION

Hypertriglyceridemia is the third most common cause of acute pancreatitis, after alcohol and gallstones. Hypertriglyceridemia accounts for 1–4% of acute pancreatitis cases [1].

Hypertriglyceridemia is variously classified as mild (150–199 mg/dL; 1.7–2.2 mmol/L), moderate (200–999 mg/dL; 2.3–11.2 mmol/L), severe (1000–1999 mg/dL; 11.2–22.4 mmol/L), and very severe (>2000 mg/dL; >22.4 mmol/L). Triglyceride levels >1,000U/L have been implicated as a cause of acute pancreatitis [2]. Types I, IV and V dyslipidemia have also been associated with acute pancreatitis. Types I and V dyslipidemia can cause acute pancreatitis without a predisposing factor, whereas Type IV can do so in the presence of an underlying condition that may increase serum triglyceride levels [3].

Poorly controlled diabetes, alcoholism, obesity, pregnancy, previous pancreatitis, and a personal or family history of hyperlipidemia should suggest the diagnosis of hypertriglyceridemic pancreatitis [4].

Conventional treatment of acute pancreatitis should be started, including, but not restricted to, aggressive hydration, analgesia and treatment of other potential causes of acute pancreatitis. In addition to such conventional management, hypertriglyceridemia needs to be treated effectively to lower triglycerides below <200 mg/dL, which has been correlated with improvement in symptoms. Infusions of insulin, heparin and plasmapheresis have been used to this effect in different subsets of patients [5, 7].

Insulin activates lipoprotein lipase, while heparin leads to the release of endothelial lipoprotein lipases. In combination, these enzymes accelerate chylomicron degradation into glycerol, and free fatty acids to lower levels of circulating lipids [5].

Lipoprotein lipase is the rate-limiting enzyme in triglyceride metabolisim, breaking it down to free fatty acids and glycerol. The free fatty acids are attached to albumin and become deactivated, otherwise they are toxic to soft tissue and could lead to local inflammation if in contact. Apheresis has emerged as an important treatment option. It could be used in patients who are normoglycemic or hyperglycemic, who failed insulin infusion. Better outcomes have been noted in patients who were plasmapheresed less that 48 hours before onset of symptoms. Complete resolution of symptoms is reported after the first round of apheresis, as long the tryglyceride levels dropped below 500 mg/dL [6, 7].

In a review of ten patients with hypertriglyceridemic pancreatitis, nine received apheresis with intravenous heparin and insulin within 48 hours of the diagnosis, with successful outcomes [6].

In general, because of its biochemical nature, hypertriglyceridemia usually triggers severe pancreatitis. Patients with severe pancreatitis may develop local or systemic complications if the condition becomes chronic. These complications are the same that could arise from severe pancreatitis due to other etiologies. Local complications of acute pancreatitis include acute peripancreatic fluid collection, pancreatic pseudocyst, acute necrotic collection, and walled-off necrosis. While acute peripancreatic fluid collections and acute necrotic collections may develop less than four weeks after the onset of pancreatitis, pancreatic pseudocyst and walled-off necrosis usually occur more than four weeks after the onset of acute pancreatitis. These have been reported with hypertriglyceridemic pancreatitis.

In summary, hypertriglyceridemia is the third most common cause of acute pancreatitis after alcohol and gall stones. The risk of hypertriglyceridemic pancreatitis is markedly increased when serum triglyceride levels exceed 1000 mg/dL and begins to increase at triglyceride levels above 500 mg/dL. Symptoms start to improve if these levels are reduced below 500 mg/dL. The aim is to reduce triglycerides to below 200 mg/dL for maintenance. If they are above 1000 mg/dL, we would recommend immediate treatment with apheresis. If apheresis is not available, the patient is hyperglycemic, or the level is less than 1000 mg/dL it is suggested that heparin and insulin be used, which leads to a compounding effect, promoting chylomicron degradation and thus a drop in triglycerides. For long-term maintenance and prevention, oral ant-hyperlipidemic agents and dietary fat restriction may be needed.

**Conflict of interest:** none declared.
